# Relationship between vertical jump performance and playing time and efficiency in professional male basketball players

**DOI:** 10.3389/fspor.2024.1399399

**Published:** 2024-06-03

**Authors:** Dimitrije Cabarkapa, Damjana V. Cabarkapa, Jelena Aleksic, Angeleau A. Scott, Andrew C. Fry

**Affiliations:** ^1^ Jayhawk Athletic Performance Laboratory—Wu Tsai Human Performance Alliance, Department of Health, Sport and Exercise Sciences, University of Kansas, Lawrence, KS, United States; ^2^ Faculty of Sport and Physical Education, University of Belgrade, Belgrade, Serbia

**Keywords:** force, strength, power, monitoring, eccentric, concentric, rebounding, shooting

## Abstract

With innovative force plate technology being available to many sports organizations worldwide that allow for time-efficient in-depth neuromuscular performance assessment, the purpose of the present study was to examine the relationship between some of the most commonly analyzed countermovement vertical jump (CVJ) force-time metrics and basketball playing time and efficiency. Twenty-four professional male basketball players volunteered to participate in the present study. The CVJ testing procedures were conducted within the first quarter of the competitive season span. Following a standardized warm-up protocol, each athlete stepped on a dual uni-axial force plate system sampling at 1,000 Hz and performed three maximum-effort CVJs with no arm swing. To minimize the possible influence of fatigue, each jump trial was separated by a 10–15 s rest interval and the average value across three jumps was used for performance analysis purposes. Basketball playing efficiency and average playing time were obtained at the end of the regular season competitive period from the coaching staff records and the official team records. Pearson product-moment correlation coefficients (*r*) were used to examine the strength of the relationships between force-time metrics and basketball playing time and efficiency, separately for each dependent variable (*p *< 0.05). A significant positive association was observed between playing efficiency and eccentric mean force and eccentric mean and peak power (*r *= 0.406–0.552). Similarly, an increase in eccentric mean power was positively correlated with the number of minutes played during the competitive season (*r *= 0.464). Moreover, the aforementioned relationship remained present even when eccentric mean power was expressed relative to the player's body mass (*r *= 0.406). Thus, the findings of the present study indicate that, at the professional level of men's basketball competition, CVJ eccentric strength and power have a positive impact on both playing time and efficiency.

## 1 Introduction

Basketball is a world-renowned sport characterized by repetitive high-intensity jumping, sprinting, and change-of-direction movements (1–3). Based on the on-court competitive demands, basketball players are required to possess adequate levels of lower-body strength and power, regardless of the playing position (4–9). Alongside tactical-technical comprehension and proficiency, these physical performance parameters are known to be of critical importance for successful participation in the game of basketball (10–13).

The countermovement vertical jump (CVJ) is a foundational movement for performing various basketball-specific skills such as shooting, rebounding, and dunking. Thus, it has been widely used by sports scientists and strength and conditioning practitioners as a non-invasive and time-efficient testing modality for the assessment of lower-body neuromuscular performance characteristics (14–19). When performed on portable force plate systems that allow rapid data analysis, a plethora of force-time metrics can be obtained with high levels of validity and reliability (16, 20). This includes metrics within both the eccentric and concentric phases of the jumping movement, which offers a comprehensive movement analysis and avoids quantifying an athlete's performance based on a single variable (e.g., jump height) (12, 18, 19). For example, a series of recently published research reports examined changes in CVJ force-time metrics pre-post practice, differences between playing positions (e.g., guards, forwards, centers), and differences between starters and non-starters at the professional level of men's basketball competition (6, 12, 21). However, it should be noted that representing force-time metrics in both relative and absolute terms may be beneficial, as heavier athletes often demonstrate superior absolute force production simply due to greater body mass (i.e., force = mass × acceleration) (6). Moreover, the same CVJ analysis approach has been used to track season-long neuromuscular performance changes in high-level collegiate basketball players (22). Overall, this type of information can help sports practitioners obtain a deeper insight into individual player's capabilities that can ultimately be used to optimize their on-court performance.

Despite being widely used in an applied sports setting, to date only a few research studies have focused on examining the relationship between CVJ performance and playing time and efficiency (8, 23, 24). When studying a cohort of National Collegiate Athletic Association (NCAA) Division-I basketball players, Hoffman et al. (8) found that greater vertical jump height was strongly correlated with playing time (*r *= 0.68). On the other hand, Lockie et al. (24) reported a weak non-statistically significant relationship (*r *= 0.22) between the same two variables of interest, suggesting that basketball-specific skills may have a larger impact on securing more playing time than CVJ performance at the NCAA Division-II competitive level. Similar findings were reported by McGill et al. (23) who found that vertical jump height had no significant relationship with the number of games played and minutes, points, and rebounds per game (*r *= 0.15–0.36), as game-related parameters used to derive player's efficiency ratings. However, the aforementioned research reports were primarily focused on studying outcome metrics such as jump height. This may be misleading in certain instances as it may fail to account for neuromuscular strategies used to attain a certain level of performance (e.g., how the specific outcome was achieved) (25, 26). Therefore, the analysis of force-time metrics within both eccentric and concentric phases of the CVJ may be advantageous in detecting differences in neuromuscular performance.

With innovative force plate technology being available to many sports organizations worldwide that allow for time-efficient in-depth neuromuscular performance assessment, the purpose of the present study was to examine the correlation between some of the most commonly analyzed CVJ force-time metrics and playing time and efficiency on the professional level of basketball competition. It is hypothesized that superior CVJ performance would be positively related to athletes' on-court playing performance (8).

## 2 Materials and methods

### 2.1 Participants

Twenty-four professional male basketball players (age = 22.7 ± 3.9 years; height = 198.1 cm ± 6.6 cm; body mass = 92.8 ± 8.9 kg; playing experience = 9.2 ± 2.1 years) volunteered to participate in the present study. All athletes competed on a similar level of basketball competition (e.g., Adriatic Basketball League) and were free of musculoskeletal injuries that could impact CVJ performance. The testing procedures performed in this investigation were previously approved by the University's Institutional Review Board and all participants signed an informed consent document.

### 2.2 Research design

The CVJ testing procedures were conducted within the first quarter of the competitive season (i.e., September–May/6–8 games after the start of the season), 48 h following the last game played during the testing week (17:00–20:00 h). Following a standardized warm-up protocol (i.e., dynamic stretching exercises and low-intensity 5–10 min of basketball partner shooting), each athlete stepped on a dual uni-axial force plate system (ForceDecks max, VALD, Brisbane, Australia) and performed three maximum-effort CVJs with no arm swing (i.e., hands on the hips during the entire movement) (11, 18). The sampling frequency of the force plate system was 1,000 Hz and the average value across three jumps was used for performance analysis purposes. To minimize the possible influence of fatigue, each trial was separated by a 10–15 s rest interval. Throughout the testing procedures, strong verbal encouragement was provided while the athletes were instructed to focus on pushing the ground as forcefully as possible (27).

### 2.3 Variables

The CVJ force-time metrics examined in the present investigation were eccentric and concentric mean and peak power [i.e., expressed in absolute and relative terms, adjusted by athletes' body mass (BM)], jump height (i.e., impulse-momentum calculation) and reactive strength index-modified (i.e., jump height divided by contraction time). The selection of the aforementioned variables was based on previously published research reports that demonstrated strong levels of validity and reliability for neuromuscular performance assessment (11, 19, 28–31). In addition, the basketball playing efficiency (Equation 1) (32) and average playing time were obtained at the end of the season (i.e., regular season competitive period) from the coaching staff records and the official team records.

Equation 1. Basketball playing efficiency formula (EFF – efficiency; PTS – points; RBD – rebounds; AS – assists; ST – steals; BL – blocks; MFG – missed field goals; MFT – missed free-throws; TO – turnovers; GP – games played).

(1) $$\mathrm{EFF}=\frac{(\mathrm{PTS}\;+\;\;\mathrm{RBD}\;+\;\mathrm{AS}\;+\;\mathrm{ST}\;+\;\mathrm{BL}-\mathrm{MFG}-\mathrm{MFT}-\mathrm{TO})\;}{\mathrm{GP}}$$

### 2.4 Statistical analysis

Descriptive statistics, means and standard deviations $\left(\bar{x}\pm \mathrm{SD}\right)$ were calculated for each dependent variable. Shapiro–Wilk test and Q-Q plots were used to assess the assumption of normality. Pearson product-moment correlation coefficients (*r*) were used to examine the strength of the relationships between force-time metrics and basketball playing time and efficiency, separately for each dependent variable. The magnitude correlation thresholds were interpreted as follows: 0.0–0.1–trivial; 0.1–0.3–small; 0.3–0.5–moderate; 0.5–0.7–large; 0.7–0.9–very large; 0.9–1.0–near-perfect to perfect (33, 34). Statistical significance was set *a priori* to *p *< 0.05. All statistical analyses were completed with SPSS (Version 26.0; IBM Corp., Armonk, NY, USA).

## 3 Results

**Figure 1:**
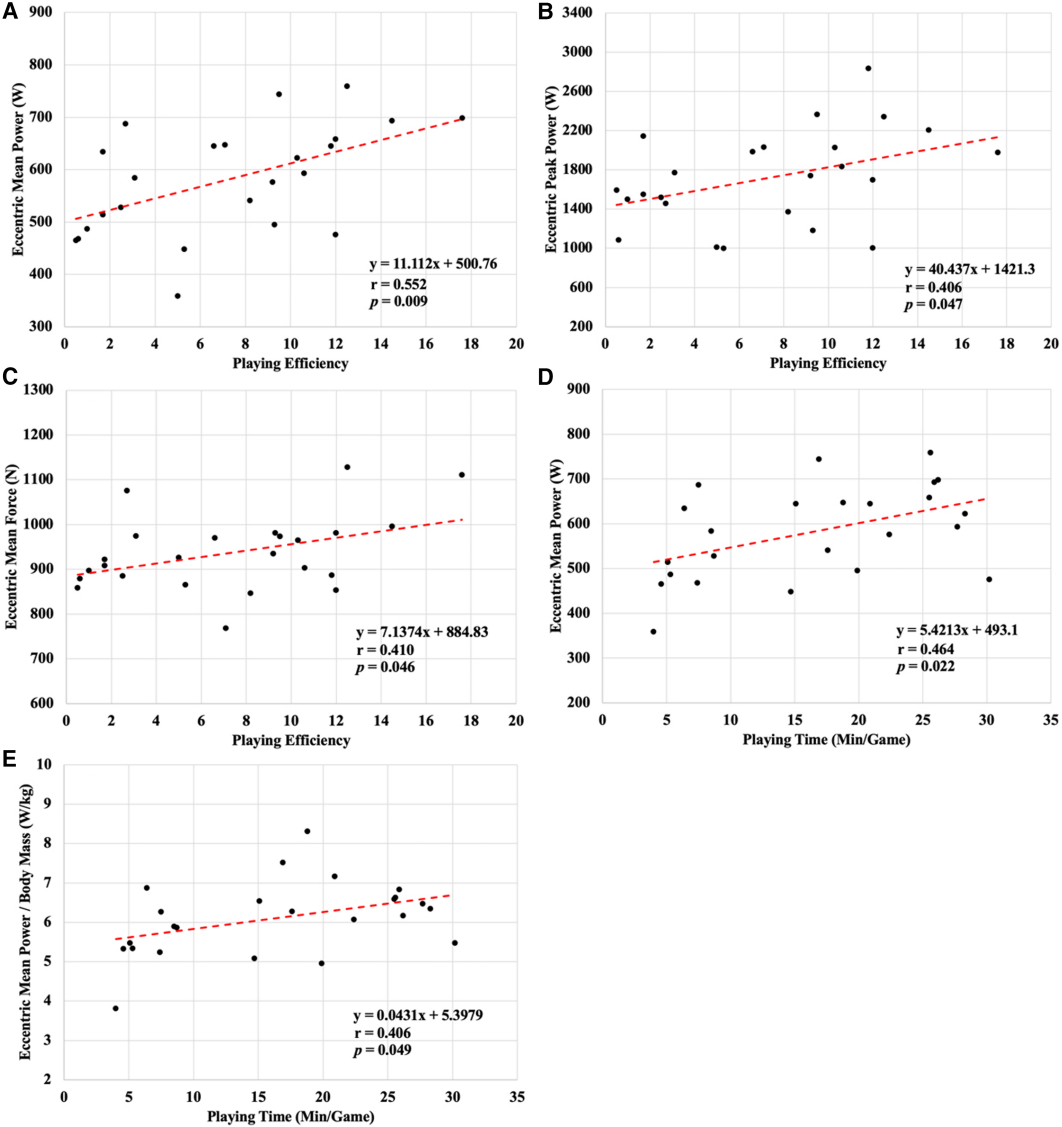
Statistically significant relationships between (**A**) eccentric mean power and playing efficiency, (**B**) eccentric peak power and playing efficiency, (**C**) eccentric mean force and playing efficiency, (**D**) eccentric mean power and playing time, and (**E**) eccentric mean power adjusted by body mass and playing time.

Descriptive statistics for each dependent variable are presented in Table 1. The average basketball playing efficiency score and playing time were 7.3 ± 4.8 and 16.3 ± 8.9 min, respectively. Statistically significant moderate relationships were found between basketball playing efficiency and eccentric mean power, eccentric peak power, and eccentric mean force. Also, a significant moderate association was observed between playing time and eccentric mean power as well as eccentric mean power/BM. No other relationships reached the level of statistical significance (*p *> 0.05) and were negligible to small in magnitude (Table 2). The graphical representation of the association between statistically significant vertical jump performance metrics and basketball playing efficiency and time is presented in Figure 1.

**Table 1:** Descriptive statistics means and standard deviations ($\bar{x}$ ± SD) for each vertical jump performance parameter.

Variable [unit]	Value
Eccentric mean power [*W*]	581.9 ± 103.9
Eccentric peak power [*W*]	1,716.7 ± 482.4
Eccentric mean power/BM [*W*/kg]	6.1 ± 0.9
Eccentric peak power/BM [*W*/kg]	18.3 ± 5.1
Eccentric mean force [*N*]	936.9 ± 84.9
Eccentric peak force [*N*]	2,279.1 ± 300.7
Eccentric mean force/BM [*N*/kg]	9.8 ± 0.1
Eccentric peak force/BM [*N*/kg]	24.0 ± 2.9
Concentric mean power [*W*]	2,930.9 ± 331.0
Concentric peak power [*W*]	5,146.6 ± 554.1
Concentric mean power/BM [*W*/kg]	30.9 ± 3.5
Concentric peak power/BM [*W*/kg]	54.2 ± 6.4
Concentric mean force [*N*]	1,969.9 ± 200.1
Concentric peak force [*N*]	2,416.9 ± 305.1
Concentric mean force/BM [*N*/kg]	20.7 ± 1.9
Concentric peak force/BM [*N*/kg]	25.4 ± 3.1
Jump height [cm]	37.9 ± 5.1
RSI-modified [ratio]	0.51 ± 0.1

BM, body mass; RSI, reactive strength index.

**Table 2:** Pearson product-moment correlation coefficients (*r*) and their statistical significance (*p*-value) between force-time metrics and basketball playing time and efficiency.

Variable [unit]	Playing efficiency		Playing time	
	*r*	*p*-value	*r*	*p*-value
Eccentric mean power [*W*]	**0**.**522**	**0**.**009**	**0**.**464**	**0**.**022**
Eccentric peak power [*W*]	**0**.**406**	**0**.**047**	0.325	0.121
Eccentric mean power/BM [*W*/kg]	0.364	0.180	**0**.**406**	**0**.**049**
Eccentric peak power/BM [*W*/kg]	0.273	0.197	0.243	0.252
Eccentric mean force [*N*]	**0**.**410**	**0**.**046**	0.235	0.270
Eccentric peak force [*N*]	0.148	0.490	0.007	0.972
Eccentric mean force/BM [*N*/kg]	0.260	0.221	0.266	0.223
Eccentric peak force/BM [*N*/kg]	0.146	0.496	0.190	0.374
Concentric mean power [*W*]	0.018	0.933	0.013	0.953
Concentric peak power [*W*]	0.025	0.906	0.053	0.806
Concentric mean power/BM [*W*/kg]	0.280	0.185	0.177	0.407
Concentric peak power/BM [*W*/kg]	0.265	0.211	0.260	0.220
Concentric mean force [*N*]	0.121	0.574	0.030	0.889
Concentric peak force [*N*]	0.105	0.625	0.067	0.758
Concentric mean force/BM [*N*/kg]	0.260	0.221	0.226	0.220
Concentric peak force/BM [*N*/kg]	0.179	0.401	0.234	0.271
Jump height [cm]	0.130	0.546	0.072	0.737
RSI-modified [ratio]	0.108	0.616	0.057	0.792

Bolded values represent statistically significant relationships (*p *< 0.05).

## 4 Discussion

To the best of our knowledge, this is the first study that focused on examining the relationship between CVJ force-time metrics and playing time and efficiency on the professional level of men's basketball competition. Overall, the findings reveal a meaningful impact of the CVJ eccentric phase on basketball on-court playing performance. A significant positive association was observed between playing efficiency and eccentric mean force and eccentric mean and peak power (*r *= 0.406–0.552). In a similar manner, an increase in eccentric mean power was positively correlated with the number of minutes played during the competitive season (*r *= 0.464). Moreover, it should be noted that the aforementioned relationship remained present even when eccentric mean power was expressed relative to the player's body mass (*r *= 0.406).

Our findings are similar to the results obtained in the previously published research reports (8, 9). Specifically, Hoffman et al. (8) found statistically significant moderate correlations between vertical jump performance and playing time (*r *= 0.58–0.68) within a cohort of NCAA Division-I male basketball players. The results of the aforementioned investigation indicated that players who had higher vertical jump values had greater playing time. Moreover, Spiteri et al. (9) emphasized the significant contribution (i.e., 25.3%) of the eccentric phase of CVJ when assessing overall player's strength profile, showing a strong association (*r = *−0.79–0.89) between eccentric strength and change-of-direction performance (i.e., *T*-test and 505 test), as commonly used testing modalities with basketball players (35, 36). This implies that the eccentric phase of the CVJ is particularly important for performing various basketball-specific movements, especially given the fact that this phase corresponds to the active stretching of leg extensor muscles which enable the body to effectively decelerate and change the movement direction (37). Furthermore, considering the well-known force-velocity relationship, increments in the velocity of eccentric muscle actions can contribute to greater force generation and higher jump heights (38, 39). As basketball players typically execute 40–50 vertical jumps per game (40, 41) and perform multidirectional movement changes approximately every 1–2 s during gameplay (10), the role of eccentric muscle actions becomes crucial for successful on-court performance, especially when considering the execution of tasks such as rebounding and blocking. Thus, it can be inferred that players who possess superior eccentric muscle qualities are likely to exhibit more efficient gameplay and ultimately secure more playing time.

While eccentric force-time metrics emerge as the predominant factors associated with basketball players' performance, it is equally important not to disregard the significance of concentric strength and power when conducting the overall assessment of basketball players. Despite no statistically significant correlations being observed in the present investigation, the development of these physical performance attributes is foundational for attaining peak levels of competition (9). For example, a recent study revealed that top-tier basketball players (e.g., NBA) possess notably higher levels of lower-body strength and power compared to college-level players (e.g., NCAA Division-I) (4). Moreover, it is well-known that maximal strength serves as a foundational piece for all other strength components, including eccentric strength (42). In a basketball-specific context, this notion is reinforced by the previously mentioned study conducted by Hoffman et al. (8), which revealed a positive correlation between maximal players' strength (i.e., barbell back squat one-repetition maximum) and playing time within a cohort collegiate basketball players. However, it should be noted that the same study also indicated that exceeding the normative levels of maximal strength did not yield additional improvements in on-court basketball performance. Thus, it is reasonable to assume that the participants in our study already had adequate levels of strength and power, particularly considering the fact that they are all already competing at the professional level of play (e.g., Adriatic Basketball League). Moreover, the observed magnitudes for both the eccentric and concentric CVJ force-time metrics fall well within the range observed in previously published research reports involving top-tier basketball players (6, 22, 39).

While the present investigation focused on examining some specific physical performance characteristics (e.g., mean and peak force and power), it is important to acknowledge that a player's excellence is a multifaceted concept. As highlighted by Hoffman et al. (8), besides vertical jump performance being one of the key predictors for securing more playing time, the best predictor was found to be the coach's evaluation of the player's competitive abilities. This notion emphasizes the fact that assessing a player's physical performance characteristics is just one piece of a large puzzle that contributes to a successful on-court basketball performance. Therefore, objective and comprehensive evaluation of players' capabilities should be always prioritized.

Despite providing additional insight into neuromuscular performance characteristics of professional male basketball players and how they relate to playing time and efficiency, this study is not without limitations. The CVJ testing procedures took place at a single time point during the in-season competitive period, suggesting that potential variations in results may be present if the testing procedures were carried out throughout different stages of the training macrocycle. Moreover, more comprehensive insight into athletes' performance might be obtained through frequent testing, perhaps on a weekly or bi-weekly basis, as this would allow sports practitioners to observe changes in various force-time metrics throughout an entire season. Last but not least, future research should also explore whether these findings are position-specific and sex-specific as well as if they remain applicable to other competitive levels (e.g., amateur, collegiate).

In conclusion, the findings of the present study indicate that, at the professional level of men's basketball competition, eccentric strength and power during a CVJ have a positive impact on both playing time and efficiency. These insights are particularly beneficial for coaches, strength and conditioning practitioners, and sports scientists, as they highlight the importance of phase-specific CVJ evaluation in order to detect differences in neuromuscular performance characteristics. Moreover, these findings provide normative ranges for this specific group of basketball players that could help sports practitioners with developing training regimens targeted toward eccentric strength and power, which may ultimately yield better overall performance of players, particularly in terms of better playing efficiency and increased playing time.

## Data Availability

The raw data supporting the conclusions of this article will be made available by the authors, without undue reservation.

## References

[1] Cormery B Marcil M Bouvard M. Rule change incidence on physiological characteristics of elite basketball players: a 10-year investigation. Br J Sports Med. (2008) 42:25–30. 10.1136/bjsm.2006.033316 17526624

[2] Hoffman JR. Handbook of sports medicine and science: basketball. In: McHeag DB, editor. Physiology of Basketball. Hoboken, NJ: Wiley-Blackwell (2003). p. 12–24.

[3] Mihajlovic M Cabarkapa D Cabarkapa DV Philipp NM Fry AC. Recovery methods in basketball: a systematic review. Sports. (2023) 11:230. 10.3390/sports11110230 37999447 PMC10675622

[4] Cabarkapa D Fry AC Lane MT Hudy A Dietz PR Cain GJ The importance of lower body strength and power for future success in professional men’s basketball. Sports Sci Health. (2020) 10:10–6. 10.7251/SSH2001010C

[5] Cabarkapa D Eserhaut DA Fry AC Cabarkapa DV Philipp NM Whiting SM Relationship between upper and lower body strength and basketball shooting performance. Sports. (2022) 10:139. 10.3390/sports10100139 36287752 PMC9611016

[6] Cabarkapa D Philipp NM Cabarkapa DV Fry AC. Position-specific differences in countermovement vertical jump force-time metrics in professional male basketball players. Front Sports Act Liv. (2023) 5:1218243. 10.3389/fspor.2023.1218234 PMC10398786 37547821

[7] Delextrat A Cohen D. Strength, power, speed, and agility of women basketball players according to playing position. J Strength Cond Res. (2009) 23(7):1974–81. 10.1519/JSC.0b013e3181b86a7e 19855320

[8] Hoffman JR Tenenbaum G Maresh CM Kraemer WJ. Relationship between athletic performance tests and playing time in elite college basketball players. J Strength Cond Res. (1996) 10:67–71. 10.1519/1533-4287(1996)010<0067:RBAPTA>2.3.CO;2

[9] Spiteri T Nimphius S Hart NH Specos C Sheppard JM Newton RU. Contribution of strength characteristics to change of direction and agility performance in female basketball athletes. J Strength Cond Res. (2014) 28:2415–23. 10.1519/JSC.0000000000000547 24875426

[10] Abdelkrim NB Castagna C El Fazaa S El Ati J. The effect of players’ standard and tactical strategy on game demands in men’s basketball. J Strength Cond Res. (2010) 24:2652–62. 10.1519/JSC.0b013e3181e2e0a3 20885192

[11] Cabarkapa D Philipp N Cabarkapa D Eserhaut D Fry A. Comparison of force-time metrics between countermovement vertical jump with and without an arm swing in professional male basketball players. Int J Strength Cond. (2023) 3:1–7. 10.47206/ijsc.v3i1.197

[12] Cabarkapa D Cabarkapa DV Philipp NM Knezevic OM Mirkov DM Fry AC. Pre-post practice changes in countermovement vertical jump force-time metrics in professional male basketball players. J Strength Cond Res. (2023) 37:609–12. 10.1519/JSC.0000000000004608 37883409

[13] Ostojic SM Mazic S Dikic N. Profiling in basketball: physical and physiological characteristics of elite players. J Strength Cond Res. (2006) 20:740–4. 10.1519/R-15944.1 17149984

[14] Heishman A Daub B Miller R Brown B Freitas E Bemben M. Countermovement jump inter-limb asymmetries in collegiate basketball players. Sports. (2019) 7:103. 10.3390/sports7050103 31052258 PMC6572434

[15] Thomas C Kyriakidou I Dos’ Santos T Jones PA. Differences in vertical jump force-time characteristics between stronger and weaker adolescent basketball players. Sports. (2017) 5:63. 10.3390/sports5030063 29910423 PMC5968968

[16] Merrigan JJ Strang A Eckerle J Mackowski N Hierholzer K Ray NT Countermovement jump force-time curve analyses: reliability and comparability across force plate systems. J Strength Cond Res. (2023) 38:30–7. 10.1519/JSC.0000000000004586 37815253

[17] Pehar M Sekulic D Sisic N Spasic M Uljevic O Krolo A Evaluation of different jumping tests in defining position-specific and performance-level differences in high-level basketball players. Biol Sport. (2017) 34:263–72. 10.5114/biolsport.2017.67122 29158620 PMC5676323

[18] Philipp NM Cabarkapa D Eserhaut DA Cabarkapa DV Fry AC. Countermovement jump force-time metrics and maximal horizontal deceleration performance in professional male basketball players. J Appl Sports Sci. (2022) 2:11–27. 10.37393/JASS.2022.02.2

[19] Philipp NM Cabarkapa D Nijem RM Blackburn SD Fry AC. Vertical jump neuromuscular performance characteristics determining on-court contribution in male and female NCAA division I basketball players. Sports. (2023) 11:239. 10.3390/sports11120239 38133106 PMC10748117

[20] Merrigan JJ Stone JD Galster SM Hagen JA. Analyzing force-time curves: comparison of commercially available automated software and custom MATLAB analyses. J Strength Cond Res. (2022) 36:2387–402. 10.1519/JSC.0000000000004275 35916879

[21] Cabarkapa D Cabarkapa DV Aleksic J Philipp NM Scott AA Johnson QR Differences in countermovement vertical jump force-time metrics between starting and non-starting professional male basketball players. Front Sports Act Liv. (2023) 5:1327379. 10.3389/fspor.2023.1327379 PMC10755471 38162698

[22] Philip NM Cabarkapa D Nijem RM Fry AC. Changes in countermovement jump force-time characteristic in elite male basketball players: a season-long analyses. PLoS One. (2023) 18:e0286581. 10.1371/journal.pone.0286581 37756277 PMC10529540

[23] McGill SM Andersen JT Horne AD. Predicting performance and injury resilience from movement quality and fitness scores in a basketball team over 2 years. J Strength Cond Res. (2012) 26:1731–9. 10.1519/JSC.0b013e3182576a76 22505125

[24] Lockie RG Beljic A Ducheny SC Kammerer JD Dawes JJ. Relationships between playing time and selected NBA combine test performance in division I mid-major basketball players. Int J Exerc Sci. (2020) 13:583. 32509125 10.70252/XZEP5232 PMC7241640

[25] Merrigan JJ Stone JD Thompson AG Hornsby WG Hagen JA. Monitoring neuromuscular performance in military personnel. Int J Environ Res Public Health. (2020) 17:9147. 10.3390/ijerph17239147 33297554 PMC7730580

[26] Cabarkapa DV Cabarkapa D Philipp NM Fry AC. Competitive season-long changes in countermovement vertical jump force-time metrics in female volleyball players. J Strength Cond Res. (2024) 38:72–7. 10.1519/JSC.0000000000004713 38258833

[27] Kershner AL Fry AC Cabarkapa D. Effect of internal vs. external focus of attention instructions on countermovement jump variables in NCAA division I student-athletes. J Strength Cond Res. (2019) 33:1467–73. 10.1519/JSC.0000000000003129 31125324

[28] Anicic Z Janicijevic D Knezevic OM Garcia-Ramos A Petrovic MR Cabarkapa D Assessment of countermovement jump: what should we report? Life. (2023) 13:190. 10.3390/life13010190 36676138 PMC9865236

[29] Bishop C Jarvis P Turner A Balsalobre-Fernandez C. Validity and reliability of strategy metrics to assess countermovement jump performance using the newly developed smartphone application. J Hum Kinet. (2022) 83:185–95. 10.2478/hukin-2022-0098 36157951 PMC9465756

[30] Heishman AD Daub BD Miller RM Freitas ED Frantz BA Bemben MG. Countermovement jump reliability performed with and without an arm swing in NCAA division I intercollegiate basketball players. J Strength Cond Res. (2020) 34:546–58. 10.1519/JSC.0000000000002812 30138237

[31] Merrigan JJ Rentz LE Hornsby WG Wagle JP Stone JD Smith HT Comparisons of countermovement jump force-time characteristics among national collegiate athletic association division I American football athletes: use of principal component analysis. J Strength Cond Res. (2022) 36:411–9. 10.1519/JSC.0000000000004173 34798642

[32] Kingsley N Amsbaugh S Papadakis Z Morgan G Boolani A. Sex moderates the fitness tests-performance index relationship in collegiate basketball: a case study. Int J Exerc Sci. (2021) 2:17.

[33] Hopkins WG. A scale of magnitudes for effect statistics (2020). 325. Available online at: http://www.sportsci. org/resource/stats/effectmag

[34] Wetmore AB Moquin PA Carroll KM Fry AC Hornsby WG Stone MH. The effect of training status on adaptations to 11 weeks of block periodization training. Sports. (2020) 8:145. 10.3390/sports8110145 33142849 PMC7693826

[35] Delextrat A Cohen D. Physiological testing of basketball players: toward a standard evaluation of anaerobic fitness. J Strength Cond Res. (2008) 22:1066–72. 10.1519/JSC.0b013e3181739d9b 18545206

[36] Wen N Dalbo VJ Burgos B Pyne DB Scanlan AT. Power testing in basketball: current practice and future recommendations. J Strength Cond Res. (2018) 32:2686–700. 10.1519/JSC.0000000000002459 29401204

[37] McMahon JJ Suchomel TJ Lake JP Comfort P. Understanding the key phases of the countermovement jump force-time curve. Strength Cond J. (2018) 40:96–106. 10.1519/SSC.0000000000000375

[38] Jaric S. Force-velocity relationship of muscles performing multi-joint maximum performance tasks. Int J Sports Med. (2015) 36:699–704. 10.1055/s-0035-1547283 25806588

[39] Sarvestan J Cheraghi M Shirzad E Svoboda Z. Experience-related impacts on jump performance of elite and collegiate basketball players: investigation on force-time curvature variables. Sport Mont. (2019) 17:23–8. 10.26773/smj.190604

[40] Abdelkrim NB El Fazaa S El Ati J. Time-motion analysis and physiological data of elite under-19-year-old basketball players during competition. Br J Sports Med. (2007) 41:69–75. 10.1136/bjsm.2006.032318 17138630 PMC2658931

[41] Scanlan A Dascombe B Reaburn P. A comparison of the activity demands of elite and sub-elite Australian men’s basketball competition. J Sports Sci. (2011) 29:1153–60. 10.1080/02640414.2011.582509 21777151

[42] Hori N Newton RU Andrews WA Kawamori N McGuigan MR Nosaka K. Does performance of hang power clean differentiate performance of jumping, sprinting, and changing of direction? J Strength Cond Res. (2008) 22:412–8. 10.1519/JSC.0b013e318166052b 18550955

